# Physiotherapy Intervention for Diabetic Foot Ulcers: A Scoping Review

**DOI:** 10.3390/diseases14050158

**Published:** 2026-04-29

**Authors:** Shinsuke Imaoka, Shohei Minata, Taisuke Teroh, Kotaro Matsuki, Ryotaro Hiramatsu

**Affiliations:** Department of Rehabilitation, Oita Oka Hospital, Nishitsurusaki 3-7-11, Oita 870-0192, Japan

**Keywords:** physiotherapy, intervention, diabetic foot ulcers, scoping review

## Abstract

Background/Objectives: Understanding the available interventions and circumstances under which physical therapy is administered to patients with diabetic foot ulcers is important to provide more evidence regarding physical therapy and associated outcomes in this population. This study aimed to investigate the scope, nature, and extent of literature on physical therapy interventions for adults with diabetic foot ulcers. Methods: Articles on physiotherapy interventions for adults with diabetic foot ulcers published up to 30 June 2024 were included. Relevant articles were identified through searches of PubMed, Scopus, MEDLINE, and the Cochrane Library databases. Opinion articles, study protocols, meeting abstracts, and articles that did not describe physical therapy interventions were excluded. Results: The systematic search identified 13 articles that met the inclusion criteria. Eleven of the 13 articles were specifically related to outpatient physical therapy. Outpatient physiotherapy included unloading gait instruction, ankle stretching instruction, progressive resistance training, and aerobic exercise. In two other cases, exercise instructions were practiced in the early postoperative period of the wound during the hospitalization period. A multidisciplinary approach aimed at improving postoperative activities of daily living was included. The main efficacy indices were the wound reduction rate, plantar pressure reduction, hemodynamics, ankle joint range of motion, walking ability, and other physical function-related parameters. Conclusions: Physiotherapy during outpatient follow-up may contribute to preventing wound deterioration and maintaining physical function in patients with stable DFUs. However, standardized protocols regarding intervention timing, exercise intensity, and wound severity remain unestablished, and interventions should be applied cautiously based on individual clinical conditions.

## 1. Introduction

An estimated 537 million people worldwide have diabetes mellitus, of whom 19–34% develop diabetic foot ulcers (DFUs) during their lifetime [1,2]. DFUs are a common complication of poorly controlled diabetes, with an annual incidence of 1.9–2%, affecting approximately 6.1 million people worldwide [3]. DFUs are common in patients with diabetes mellitus. For diabetic patients with active ulcers, the final cure rate for hospitalization is 65–75%, whereas approximately 15–20% of all patients with ulcers undergo amputation, depending on the follow-up period [4]. In addition, treatment costs range from $18,600 to $35,100 per DFU, with $60 billion spent annually in the United States on lower extremity-related treatment of patients with diabetes [5]. Thus, the development and amputation of DFUs are associated with substantial morbidity and mortality, decreased quality of life and productivity, and considerable healthcare costs.

Kinematic factors, e.g., joint range of motion [6,7] and gait style [8], are closely associated with the development of DFUs. Severe cases of diabetic foot ulceration that lead to amputation were associated with decreased daily activities and a higher risk of death [9]. Therefore, ulcer management and its prevention may reduce complications and death [10]. Based on this background, it can be inferred that there is a great need for physical therapy in patients with DFUs, both for the prophylactic aspect of ulcers and for the maintenance of postoperative quality of life [11]. However, regarding physiotherapy interventions for DFUs, there is no selection of patients or clear intervention methods, and recommendations for patients are limited by the guidelines [12]. Additionally, the patients and background factors for whom physiotherapy is provided vary. Although a certain level of control over the patients is necessary to implement standard physiotherapy interventions, there are many unclear points [13,14].

This scoping review aimed to summarize the implementation of physical therapy to improve physical function in patients with DFUs and to identify areas for further investigation and clarification. What is the optimal timing of physical therapy for patients with DFUs? What is the content of the physical therapy program? What is the target population’s basic demographic range? What indicators are used to determine the effectiveness of a physical therapy program?

## 2. Materials and Methods

This scoping review was conducted and reported in accordance with the Preferred Reporting Items for Systematic Reviews and Meta-Analyses Extension for Scoping Reviews (PRISMA-ScR), which served as the methodological and reporting framework for this study [15,16]. The review protocol was not prospectively registered. We constructed our protocol by applying a four-step process: identifying the research question, identifying the studies, selecting the studies, and extracting and analyzing the data.

### 2.1. Step 1: Identify the Research Question

This scoping review aimed to identify a comprehensive methodology for physiotherapy intervention for patients with DFUs by systematically mapping studies that documented the indications for physiotherapy intervention for such patients. We used the PCC to identify the research questions. In this study, the research questions were formulated based on the PCC (Participants, Concept, and Context) framework. The Participants were adult patients with diabetic foot ulcers; the Concept included rehabilitation, physical therapy, and various exercise interventions; and the Context encompassed a range of settings from acute to chronic care, both in Japan and internationally. To ensure reproducibility, detailed search strings are provided in the Appendix A.

### 2.2. Step 2: Identify Relevant Studies

We used PubMed, Scopus, PEDro, MEDLINE, and the Cochrane Library databases using combinations of keywords related to ‘diabetic foot ulcer’ and ‘rehabilitation’, including physical therapy. In addition, a manual search (hand-searching) was conducted by reviewing the reference lists of identified studies to ensure a comprehensive capture of relevant literature. The last search date was 30 June 2024. Free-text terms and Boolean operators (and/or) were used when searching titles and abstracts. No filters or restrictions were applied. To ensure a comprehensive search, clinical trial registries (ClinicalTrials.gov) and gray literature were explored in addition to the four primary databases. The same search keywords and Boolean operators were applied as those used for the main databases to confirm the presence of ongoing trials and unpublished reports. These keywords were selected to cover studies that applied physiotherapy in patients with DFUs. The search strategies used in each database are listed in Table 1.

### 2.3. Step 3: Selection of Studies

Articles meeting the inclusion criteria were selected from English-language publications, and all study designs were included. This scoping review focuses on physiotherapy interventions for patients with active DFUs or those who have undergone surgical procedures, such as amputations, resulting from DFUs. Studies involving broader populations, such as those with diabetic peripheral neuropathy, were included if they provided specific data on a subgroup of patients with active DFUs or post-operative DFU cases that met our primary inclusion criteria. During the data extraction process, we specifically isolated outcomes related to the DFU or post-operative subgroups from these broader studies to ensure the findings remained focused on our primary objective. If studies did not provide disaggregated data for the DFU population, they were excluded to maintain the specificity of our analysis. Conversely, studies aimed solely at fall prevention due to neuropathy or the prevention of high-risk feet without active ulceration were excluded. Five authors used the Rayyan literature screening software (https://www.rayyan.ai/, accessed on 22 April 2024) to select eligible articles. For each article, the first author (S.I.) and two other authors (S.M., K.M., T.T., or R.H.) independently read the title and abstract to exclude irrelevant articles, and then they read the full text to see if it met the eligibility criteria. Any disagreements during the screening process were resolved through discussion among the authors or, when necessary, via evaluation by an independent third party.

### 2.4. Step 4: Data Extraction and Analysis

We extracted information on the authors, year of publication, study design, study country, patient demographics, timing of physical therapy intervention, intervention content, intervention duration, and measures of effectiveness from the eligible articles.

## 3. Results

### 3.1. Study Characteristics

A total of 722 records were initially identified through database searching (PubMed, *n* = 512; Scopus, *n* = 173; Cochrane, *n* = 12; PeDro, *n* = 8) and citation searching (*n* = 17). After removing 124 duplicate records, 598 records were screened based on titles and abstracts, resulting in the exclusion of 555 records.

**Figure 1:**
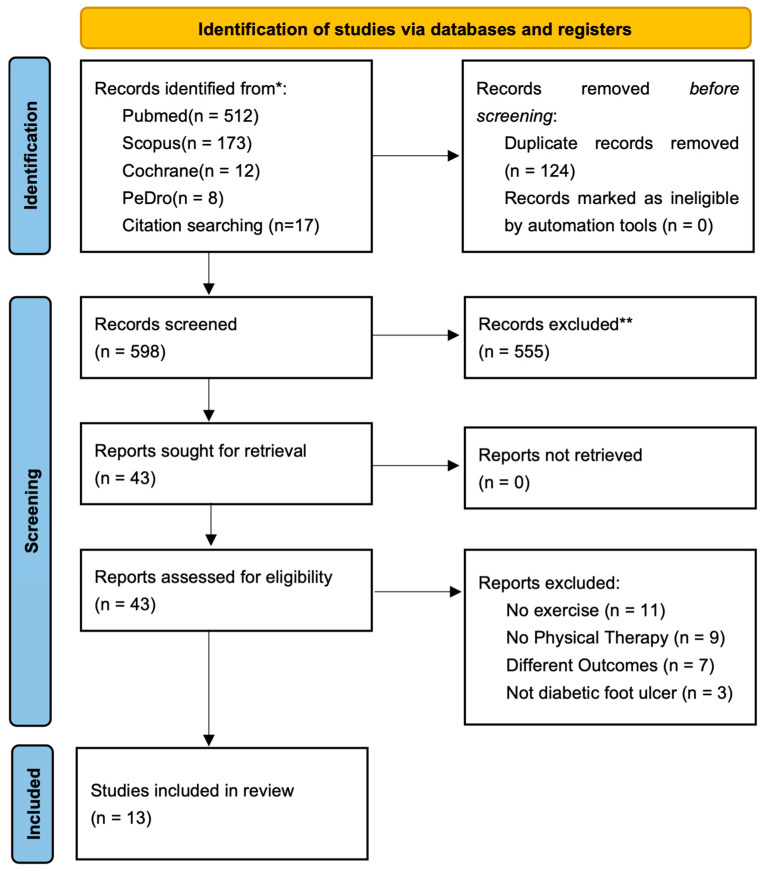
Preferred Reporting Items for Systematic Reviews and Meta-Analyses flowchart.

The remaining 43 reports were sought for retrieval, and all (*n* = 43) were successfully retrieved for full-text assessment. Upon eligibility assessment, 30 reports were excluded for the following reasons: lack of exercise intervention (*n* = 11), absence of physical therapy (*n* = 9), different outcomes (*n* = 7), and focus on conditions other than diabetic foot ulcers (*n* = 3). Finally, 13 studies met the inclusion criteria and were included in this review (Figure 1). The included studies reported a direct impact of physical therapy interventions on various clinical outcomes, including wound healing, peripheral circulation, and functional mobility (e.g., muscle strength and gait independence).

### 3.2. Studies Related to Physical Therapy Interventions for Physical Function

We identified 13 studies that contained important data on the direct effects of physical therapy interventions on the improvement of physical function in patients with DFUs. Thirteen studies were included (Figure 1). The study designs included five randomized controlled trials, three prospective cohort studies, three pre/post comparisons, and two case series. Different physical therapy interventions, durations, and outcomes were used in these studies (Table 1).

The identified studies demonstrated clear variation according to clinical settings and patient needs. Most studies (*n* = 11) focused on outpatient physiotherapy, primarily involving self-exercise instructions such as offloading gait training, ankle stretching, and resistance training. In contrast, a smaller subset (*n* = 2) addressed intensive inpatient rehabilitation during the early postoperative period to improve activities of daily living.

Intervention approaches also varied according to the underlying pathology. Studies involving patients with peripheral artery disease (PAD) predominantly utilized Buerger’s exercises to improve circulation, while those focusing on diabetic peripheral neuropathy (DPN) emphasized balance and resistance training to address functional limitations.

To improve clarity, outcomes were grouped into three primary domains. In the wound-related domain, eight of the 13 studies used wound reduction or healing rates as a primary endpoint. Consistently, evidence suggests that exercise therapy with appropriate offloading does not impede healing and may even promote wound area reduction. In the functional and physiological domain, common patterns observed included improvements in ankle range of motion, muscle strength, and walking independence. Additionally, physiological markers such as hemodynamics (measured via NIRS or SPP) showed significant improvement following specific exercise protocols. In the patient-reported and quality of life domain, early rehabilitation interventions were found to contribute positively to health-related quality of life (HRQoL), as evidenced by improved EQ-5D-5L scores.

Overall, these findings consistently indicate that physiotherapy incorporating offloading can maintain physical function without adversely affecting wound healing.

### 3.3. Distribution of Papers by Year

**Figure 2:**
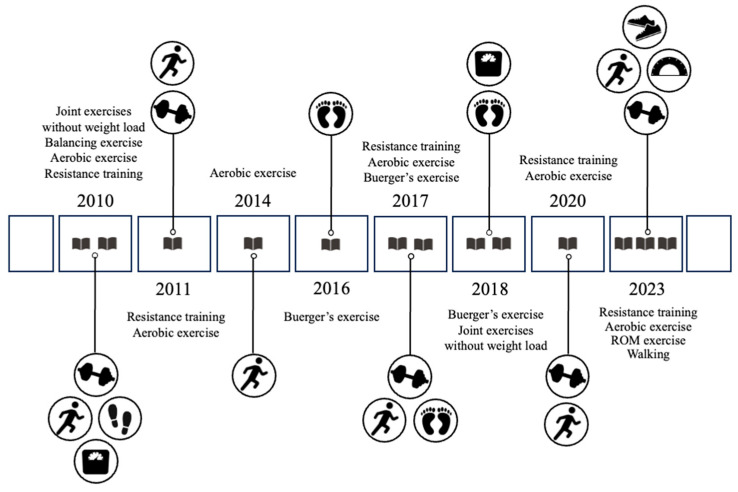
Distribution of papers by year of publication. ROM, range of motion.

In recent years, the number of published studies on physical therapy for DFUs has increased, with 69% of the reviewed studies published since 2015 (Figure 2).

### 3.4. Distribution of Papers Published by Country

The 13 studies included in the review were published by the first authors in nine countries (Table 2). Taiwan (*n* = 3), Japan (*n* = 2), Denmark (*n* = 2), Nigeria (*n* = 1), Thailand (*n* = 1), the United States (*n* = 1), Canada (*n* = 1), the Netherlands (*n* = 1), and Turkey (*n* = 1) made the largest contributions to the number of studies.

### 3.5. Evaluating the Effectiveness of Physical Therapy

**Figure 3:**
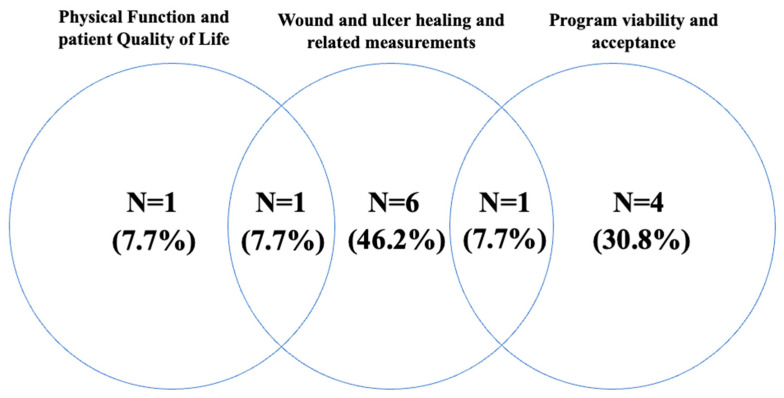
Determining the effectiveness of physical therapy.

Of the 13 studies, eight had wound-related primary endpoints, e.g., the wound reduction rate. The remaining five studies focused on physical function, quality of life, and adherence to physical therapy (Figure 3). These studies focused on observing whether physical or exercise therapy interventions helped improve DFU wounds or whether physical therapy interventions affected physical function.

## 4. Discussion

The purposes of this scoping review were to systematically map studies on physical therapy interventions aimed at improving physical function in patients with DFUs and to provide a comprehensive index of the physical therapy methodologies used to date. Our main findings are as follows.

### 4.1. Predominance of Physiotherapy Interventions During the Outpatient Follow-Up Phase

In most studies, physiotherapy was provided during the outpatient follow-up period after the wound was stabilized [16,17,18,19,20,21]. Therefore, physiotherapy is identified as a potential intervention strategy for wound management and maintenance of physical function in patients with DFUs and stable wounds. The physiotherapy intervention strategy focused on preventing wound deterioration and reducing severity during outpatient follow-up. However, few studies examined the impact of physical therapy on physical function during the wound healing period [6]. Physiotherapy may not be provided during hospitalization because of pain associated with wound treatment, postoperative infection control, and the physiotherapy implementation system. In most cases, unloading management is required during hospitalization, and the risk of loss of walking ability is high with this strategy. Therefore, research on early postoperative physiotherapy is warranted to further explore the feasibility of introducing the physiotherapy intervention during the wound healing period at an appropriate time. Future research should focus on the effectiveness of early postoperative physiotherapy interventions.

### 4.2. Diversity in Intervention Content and Exercise Load Settings Focusing on Non-Weight-Bearing Exercises

There were no guidelines or clear standards in any of the studies regarding the content of the physical therapy program, and the timing, duration, and length of the intervention differed across a wide variety of situations. Specifically, outpatient physical therapy mainly included non-weight-bearing exercises for the triceps muscle and antigravity exercises around the ankle joint with the patient in the supine and seated positions, including Buerger’s exercise [17,22,23]. The amount of exercise included various exercise load settings, ranging from 10 sets twice a day to 50 min three times per week [23,24]. In contrast, the amount of exercise was not set at any time, such as 10 sets twice a day, but was set at 50 min three times per week [23,24]. However, in an intervention study by Maeshige et al. in which physiotherapy was performed early in the wound healing period, physiotherapy was performed with the wound protected (complete unloading) five times per week for approximately 40 min for approximately 1 month from the early postoperative period, indicating that walking ability could be maintained through early unloading [25]. Regarding the intensity of exercise during hospitalization, the treatment side included many interventions that minimized the intervention in the affected lower extremity, including muscle loading around the wound in a completely unloaded state. Against this background, a systematic review of exercise during wound care showed the importance of starting walking practice with unloading and considering wound deterioration [6]. In the future, it is important to cooperate with basic researchers to establish standardized intervention criteria for teaching movement with unloading and to examine what kind of exercise load setting is most effective from the kinematics viewpoint.

### 4.3. Target Demographics Characterized by Older Male Populations and Variable Comorbidities

The participants who received physiotherapy ranged in age from their 40–80s, and the cohort included many older men who underwent both outpatient and inpatient interventions. In the case of outpatient follow-up, half of the patients had inactive ulcers, and a high percentage of the patients maintained walking [16,17,18,19,20,21]. In contrast, the patients who underwent physiotherapy during the hospitalization period were mainly those who underwent small amputations of the foot and those who required strict load-bearing management [25]. Although physiotherapy was provided from the early postoperative period to patients with severe wounds, e.g., those after small amputations, there were no adverse events [26]. Regarding diabetic complications, some patients had nephropathy and retinopathy, and the severity of the complications included various grades. However, studies focusing on children and patients with type 1 diabetes, which we believe is an unexplored area, were not included. These results suggest that the patients were mainly older men who underwent outpatient physiotherapy and that validation that controls for complications and background factors is necessary.

### 4.4. Multifaceted Outcome Measures Utilizing Wound Reduction Rates and Physical Functional Indices

Most studies used the rate of reduction in the size and area of the wound as an index for determining the effectiveness of physical therapy [16,19,28]. Other indicators included patients’ hemodynamics before and after exercise [18,23], walking ability [25,26], plantar load [24], standing balance ability [21], quality of life [25], and adherence [28]. In physical therapy, hemodynamics, walking ability, and range of motion of the foot joints are associated with wound development and wound severity [29,30]. Additionally, plantar load is an important factor in determining wound severity and may be an effective indicator for standardizing the effectiveness of physical therapy. However, the range of motion of the foot joints and plantar loading tend not to be considered as major endpoints because it is difficult to identify a clear cutoff level for the range of motion and plantar loading owing to the variety of measurement positions and measurement devices. This suggests that there is a need to select evaluation items that consider the timing of evaluation, measurement methods, and equipment to standardize these indicators and judge the effectiveness of physiotherapy in the future.

### 4.5. Study Limitations

One limitation of this study is that it is a scoping review, and we did not formally assess the specific benefits and drawbacks of each physical therapy intervention. Therefore, we cannot draw definitive conclusions regarding the effects of these interventions on the physical function of patients with DFUs. Furthermore, the review revealed substantial heterogeneity in intervention content, timing, and exercise load settings for DFU patients, reflecting variability in the current evidence base. Many included studies had methodological limitations and were of generally low quality; thus, the results must be interpreted with caution. Consequently, no definitive conclusions can be drawn regarding the overall clinical efficacy of physiotherapy in this population. To overcome these limitations, future high-quality research and closer collaboration between basic and clinical researchers are essential to establish standardized physiotherapy protocols and strengthen the evidence base. Additionally, although each study was reviewed by five experienced physical therapists, it is possible that different reviewers might have reached different conclusions.

### 4.6. Clinical Significance

The value of this study lies in its ability to complement existing limited guidelines by visualizing the specific realities of physical therapy interventions for patients with DFUs. In particular, the findings describe the current landscape of early postoperative intervention, providing a basis for discussing its possible role in maintaining walking function that serves to reinforce the critical importance of acute-phase rehabilitation. Furthermore, the heterogeneity of intervention methodologies and the challenges regarding evaluation indicators identified through this review provide a direct roadmap for the future development of standardized clinical protocols.

## 5. Conclusions

In this study, we found that the content of physiotherapy for patients with DFUs, timing of physiotherapy, and setting of the exercise load varied. Thus, it will be important to accumulate more data through cooperative research between basic and clinical researchers and to standardize the physiotherapy methods for patients with DFUs identified herein.

## Figures and Tables

**Table 1:** Summary of physiotherapy interventions for diabetic foot ulcers (DFUs).

Reference (Year)	Design	Sample Size	DFU Characteristics	Setting	Intervention Content	Frequency/ Duration	Comparison	Primary Results
Eraydin [16]	Prospective, RCT	65	Wagner grade 1 or 2 ulcers	Outpatient	Standard care + daily foot exercises	Daily/12 weeks	Standard wound care only	Significant decrease in ulcer area and depth in the intervention group.
Otterman [17]	Pre-post design study	22	Diabetes with physical limitations in ADL	Outpatient	Individualized aerobic and resistance exercise	12 weeks	Pre- vs. post-intervention	High adherence; significant improvements in HbA1c, muscle strength, and perceived function.
Chang [18]	Observational study	30	Unilateral or bilateral diabetic foot ulcers	Outpatient	Buerger’s exercise with SPP measurement	3 times daily/3 months	Pre- vs. post-intervention	Significant increase in SPP; 27% of ulcers completely healed, 41% improving.
Lindberg [19]	Case series	5	Severe neuropathy and active foot ulcers	Outpatient	Non-weight-bearing aerobic and resistance training	10 weeks	Pre- vs. post-intervention	High adherence and satisfaction; reduced wound size and improved muscle strength/ADL.
Aagaard [20]	Case series	3	Diabetic foot ulcers	Outpatient	Moderate-intensity aerobic (bike) and resistance exercise	30 min/12 weeks	Pre- vs. post-intervention	Relevant and well-received, but low recruitment limited feasibility conclusions.
Kruse [21]	Observer-masked, RCT	79	Sedentary adults with DM-PN	Outpatient (Home)	Leg strengthening, balance, and walking program	12 months	Diabetes self-care instruction only	Minimal effect on balance/strength; no increase in fall risk from the exercise program.
Chen [22]	Prospective study	30 (+15 healthy)	DFU categorized by presence of PAD	Outpatient	Buerger’s exercise with NIRS monitoring	Stage-based	Healthy control group	Buerger’s exercise increased peripheral HbO_2_ and total Hb circulation.
Lin [23]	Prospective cohort study	14	Vasculopathic DFU (with/without PTA)	Outpatient (Home)	Buerger’s exercise with wireless NIRS	3 times daily/≥8 weeks	Pre- vs. post-intervention	Significant improvement in peripheral circulation and wound condition.
Kanchanasamut [24]	Controlled clinical trial	21	Diabetic peripheral neuropathy (DPN)	Outpatient	Mini-trampoline exercise + foot-care education	8 weeks (20-week follow-up)	Education only group	Improved range of motion and vibration perception; trend for decreased peak plantar pressure.
Maeshige [25]	Multicenter RCT	60	Chronic foot wounds undergoing surgery	Inpatient	Early vs. late rehabilitation sessions	5 times/week/until discharge	Late rehabilitation group	Early group maintained higher FIM-gait scores during hospitalization; QOL improved in both.
Sonoda [26]	Multicenter retrospective cohort study	232	Hospitalized patients with chronic LE wounds	Inpatient	Gait independence comparison by amputation level	During hospitalization	Comparison between amputation levels	Lisfranc amputation negatively affected gait independence compared to distal amputations.
Goddy [27]	RCT	61	Diabetic foot ulcers	Outpatient	Supervised aerobic exercise (bicycle ergometer)	60–85% max HR/12 weeks	Routine treatment only	Significant reduction in wound size and fasting plasma glucose levels.
Flahr [28]	Prospective, quasi-experimental pilot study	19	Older adults with diabetic foot wounds	Outpatient	Non-weight-bearing ankle exercises	12 weeks	Control group	No significant difference in wound reduction; 70% adherence suggests feasibility for future trials.

Abbreviations: DFU, diabetic foot ulcer; RCT, randomized controlled trial; PAD, peripheral arterial disease; PTA, percutaneous transluminal angioplasty; NIRS, near-infrared spectroscopy; SPP, skin perfusion pressure; ADL, activities of daily living; DPN, diabetic peripheral neuropathy; DM-PN, diabetes mellitus with peripheral neuropathy; LE, lower extremity; FIM, functional independence measure; QOL, quality of life; HbA1c, glycated hemoglobin; HR, heart rate.

**Table 2:** Geographical distribution of included studies.

Country	Number of Studies (*n*)	Percentage (%)
Taiwan	3	21.4%
Japan	2	14.3%
Denmark	2	14.3%
Nigeria	1	7.1%
Thailand	1	7.1%
United States	1	7.1%
Canada	1	7.1%
Netherlands	1	7.1%
Turkey	1	7.1%

## Data Availability

The datasets generated and/or analyzed in this study are available from the corresponding author upon reasonable request.

## Abbreviations

The following abbreviation is used in this manuscript:

DFU	Diabetic foot ulcer

## Appendix A. Search Terms and String

The following search string was employed to identify relevant studies:

(“diabetic foot ulcer” OR “diabetic foot lesion” OR “chronic foot wound” OR “lower extremity wounds” OR “lower-extremity ulcers” OR “plantar ulcer”) AND (“physical therapy” OR “physiotherapy” OR “physiotherapist” OR “physical therapist” OR “rehabilitation” OR “podiatrist”)
